# Effect of Protein–Protein Interactions on Translational Diffusion of Spheroidal Proteins

**DOI:** 10.3390/ijms23169240

**Published:** 2022-08-17

**Authors:** Aleksandra M. Kusova, Aleksandr E. Sitnitsky, Vladimir N. Uversky, Yuriy F. Zuev

**Affiliations:** 1 Kazan Institute of Biochemistry and Biophysics, FRC Kazan Scientific Center, Russian Academy of Sciences, Lobachevsky Str., 2/31, 420111 Kazan, Russia; 2 Department of Molecular Medicine and Byrd Alzheimer’s Research Institute, Morsani College of Medicine, University of South Florida, 12901 Bruce B. Downs Blvd., MDC07, Tampa, FL 33612, USA

**Keywords:** protein–protein interactions, collective diffusion, self-diffusion, DLVO theory, Vink theory, spheroidal proteins

## Abstract

One of the commonly accepted approaches to estimate protein–protein interactions (PPI) in aqueous solutions is the analysis of their translational diffusion. The present review article observes a phenomenological approach to analyze PPI effects via concentration dependencies of self- and collective translational diffusion coefficient for several spheroidal proteins derived from the pulsed field gradient NMR (PFG NMR) and dynamic light scattering (DLS), respectively. These proteins are rigid globular α-chymotrypsin (ChTr) and human serum albumin (HSA), and partly disordered α-casein (α-CN) and β-lactoglobulin (β-Lg). The PPI analysis enabled us to reveal the dominance of intermolecular repulsion at low ionic strength of solution (0.003–0.01 M) for all studied proteins. The increase in the ionic strength to 0.1–1.0 M leads to the screening of protein charges, resulting in the decrease of the protein electrostatic potential. The increase of the van der Waals potential for ChTr and α-CN characterizes their propensity towards unstable weak attractive interactions. The decrease of van der Waals interactions for β-Lg is probably associated with the formation of stable oligomers by this protein. The PPI, estimated with the help of interaction potential and idealized spherical molecular geometry, are in good agreement with experimental data.

## 1. Introduction

Diffusion is one of the fundamental physical phenomena characterizing functional properties of molecules and their interaction with environment [1,2,3,4,5,6,7]. Molecular diffusion is the inevitable component of specific recognition of cell community [8]. Translational diffusion is the main way of molecular transport in organisms that defines numerous vital activities of the living systems. Previously, the knowledge of protein diffusion was mainly utilized for estimation of the protein hydrodynamic dimensions under different conditions or for evaluation of molecule association. In such systems, the diffusional process is well described by the classical Stokes–Einstein model [9,10,11,12]:

(1) $$D=\frac{k_{B}T}{6\pi \eta R_{h}},$$

where *D* is the diffusion coefficient, *k_B_* is the Boltzmann constant, *T* is the temperature, *η* is the solution dynamic viscosity, and *R_h_* is the hydrodynamic radius of a particle approximated as a sphere.

**Figure 1:**
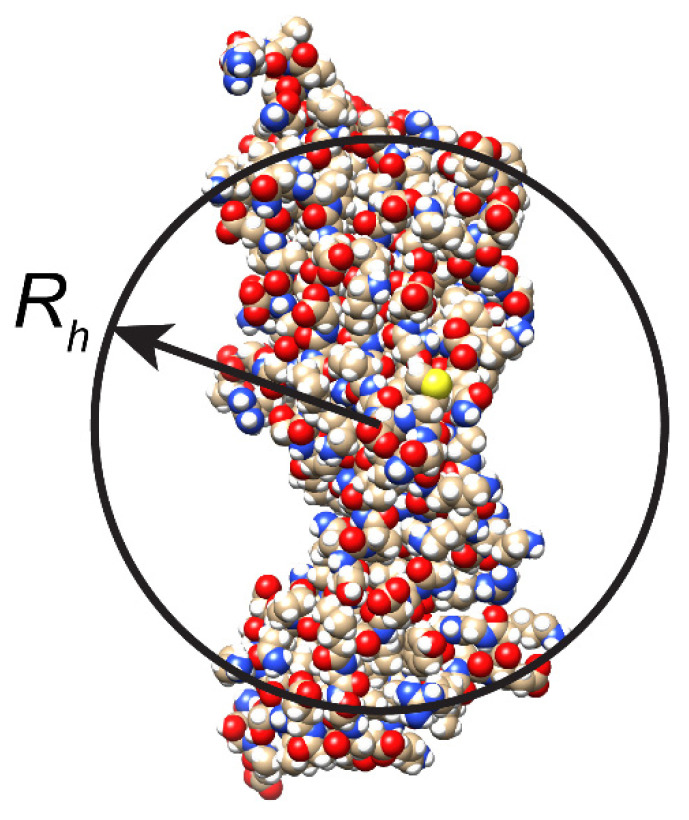
Schematic representation of effective hydrodynamic radius *R_h_* of non-spherical protein molecule.

Although the Stokes–Einstein relation was designed for rigid spheres in isotropic medium, it is often used to estimate the size of complicated biological molecules. Unfortunately, frequently, the deviation from the strict spherical shape is substantial, and the use of Stokes–Einstein relation can lead to large inaccuracies and/or misleading conclusions. Additional parameters were introduced to take into account the deviation from the protein spherical shape, i.e., ellipsoid, polymer, rod, or disk [13,14,15,16]. However, the related empirical formulas work adequately only in very limited cases. For objects of unknown shape, the size obtained from the Stokes–Einstein equation should be considered as the effective hydrodynamic radius *R_h_* (Figure 1).

In real living systems, translational diffusion of macromolecules significantly deviates from the classical representation of the diffusion in diluted aqueous solutions [17,18,19,20,21]. The internal environment of a living cell is densely crowded with macromolecules, which create steric barriers for diffusing particles. Living systems contain various types of biological macromolecules, such as DNA, RNA, proteins, and polysaccharides, all engaged in a multitude of specific and non-specific intermolecular interactions [22]. Thermodynamic heterogeneity and many other factors that exist in the cell affect protein diffusion, providing ambiguous diffusion coefficients [23,24]. In fact, due to the existence of such macromolecular obstacles (or other cellular components) that represent the so-called “cell” effect, the diffusion coefficient of molecules can exhibit anomalous behavior [23,25]. These deviations from the classical view (diluted solutions) cause the limitations of common theoretical and experimental approaches, which are mainly used to study protein dynamics [26,27]. There were many attempts to characterize non-specific intermolecular interactions in terms of excluded volume and restricted motion of studied molecules [28,29,30,31,32,33]. However, the experimental diffusion data show significant deviation of protein diffusion under crowding conditions from the phenomenological predictions [34,35].

In concentrated/crowded solutions, the non-linear behavior of the diffusion coefficient under the conditions of increased protein concentration was shown [36,37,38]. Such a trend indicates the presence of a significant deviation of protein diffusion from the Stokes–Einstein behavior in concentrated/crowded systems. It was suggested that translational diffusion of macromolecules in crowded environment differs significantly from dilute solution due to the huge number of intermolecular contacts [39,40,41,42]. The physical consequence of macromolecular crowding declares itself mainly in the hard-core repulsions and the so-called “soft” interactions [43]. The hard-core repulsion represents a steric effect arising from the impenetrable nature of atoms, which reduces the available free volume for their diffusive motion. The “soft” interactions include hydrogen bonding, charge–charge, solute–protein, van der Waals, and hydrophobic interactions [44]. Of these, only the strong electrostatic repulsion of similarly charged molecules prevents their convergence. Other “soft” interactions are attractive and destabilizing, because they favor the expanded conformations that allow the access to attractive surfaces. The effect of intracellular environment modulating protein–protein interactions (PPI) is important because the totality of weak interactions in the cells forms the crowded cellular interior [45,46,47]. The simplest systems for modeling the intermolecular interactions of proteins in cells are solutions with one type of macromolecules at different concentrations [48]. Therefore, one of the well-known ways for estimation of the intermolecular interactions of proteins in aqueous solutions is the analysis of their translational diffusion in a wide concentration range [49,50].

Earlier, we have shown that the PPI estimation can be based on comparative analysis of self- and collective translational diffusion [51,52]. The technique of pulsed field gradient nuclear magnetic resonance (PFG NMR) operates on the experimental time scales exceeding those of the intermolecular collisions. The long-time self-diffusion coefficient *D_s_* is observed as the averaged result of protein diffusivity over a long observation time [3,53,54,55,56,57]. The value of *D_s_* can be characterized by the Stokes–Einstein equation via the coefficient of protein hydrodynamic friction *f*_12_ with solvent molecules [58]:

(2) $$D_{s}=\frac{kT}{f_{12}}.$$

This relationship is correct only for spherical proteins in dilute homogeneous solution. The Stokes–Einstein equation was found to well-describe the variety of different systems, such as hard sphere dispersions [59,60], microemulsions [61], micellar solutions [62], and protein dispersions [63]. However, for solutions of charged particles in semi-diluted and concentrated solutions, the strong deviation from the Stokes–Einstein relation was experimentally observed [38,64]. When the protein volume fraction *φ* increases, the PPI growth results in an additional friction term *f*_22_(*φ*) in the Stokes–Einstein relation. *f*_22_(*φ*) is a phenomenological parameter introduced to describe the self-diffusion slowing down due to the increase in the “local viscosity”. Thus, one can write [65]:

(3) $$D_{s}\left(\varphi \right)=\frac{kT}{f_{12}+f_{22}\left(\varphi \right)}.$$

Such an interpretation of the Stokes–Einstein relation successfully describes the behavior of proteins self-diffusion coefficient in semi-dilute and concentrated solutions [66,67,68].

In dilute solutions, the molecules move independently of each other, whereas in the semi-dilute solutions, the intermolecular interactions result in appearance of new class of motion-collective modes. It is described by the Fick law [69,70]:

(4) $$j(r,t)=-D_{c}\nabla \rho (r,t)$$

where *D_c_* is the collective diffusion coefficient, **j** is the diffusion flux vector, *ρ* is the instantaneous number density (number of molecules per unit volume) at position **r** and time *t*.

The collective diffusion coefficient *D_c_* takes into account the local small-displacement mobility of a tracer particle in the medium at equilibrium [71]. It depends on the microscopic fluctuations in the local concentration of particles and the corresponding local inhomogeneity in the refractive index of medium [72]. The technique of dynamic light scattering (DLS) is sensitive to the local fluctuations of particle concentration and provides the means for measurements of the short time collective diffusion coefficient *D_c_.*

**Figure 2:**
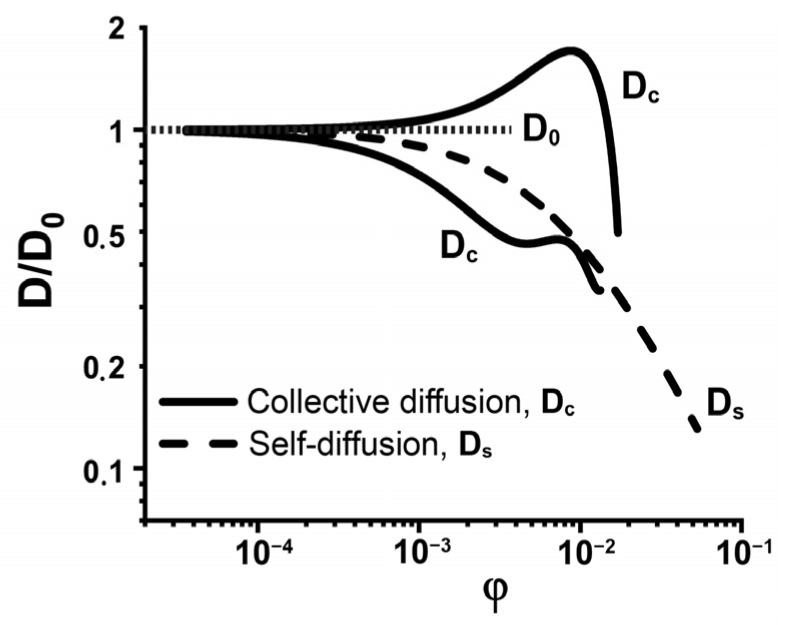
Schematic representation of protein concentration dependence of collective (*D_c_*) and self-diffusion (*D_s_*) coefficients, *D_c_* in the cases of the repulsive and attractive inter-molecular interactions, *D*_0_ is diffusion coefficient in dilute solution. The original data were obtained from [52].

For dilute systems, where inter-molecular interactions and resulting deviations from the average density are absent, the diffusion coefficient is a constant. In the case of dilute solutions, the self- and collective diffusion coefficients are identical *D_c_* = *D_s_* = *D*_0_, where the subscript “0” indicates that interactions between diffusing species are absent. In practice, in most of systems, the concentration of diffusing molecules is noticeable, and the inter-molecular interactions affect the translational diffusion [24]. At the intermediate concentrations, *D_c_* and *D_s_* differ from *D*_0_ and strongly depend on the inter-molecular interactions (Figure 2) [49,73]. It was shown [52,74,75] that the collective diffusion coefficient *D_c_* tends to increase with the growth of the repulsive interactions between molecules and decrease with the prevalence of attractive ones (Figure 2).

The concentration dependencies of the protein self- and collective diffusion coefficients contain information about the contributions from various intermolecular interactions [76,77,78]. Weak PPI are commonly characterized in terms of virial coefficients [49,79,80,81] and the friction formalism [82,83], providing the linkage between solvent- and solute-mediated interactions. To date, the studies of PPI in dilute solutions are limited by the second (paired) virial coefficient *A*_2_ [79,80], which is inter-related to the paired interaction potential determined by the Deryaguin–Landau–Verwey–Overbeek (DLVO) theory [84,85]. The DLVO theory was applied to describe interactions between biological colloids, such as cells, vesicles, and micelles [86,87]. Recently, we have proposed a complex approach to study the interactions of protein molecules via the analysis of their diffusive mobility over a wide concentration range [51]. The next subsection overviews the main points of the Vink theory, which provides an explicit expression for the self- and collective diffusion coefficients in terms of the basic principles of non-equilibrium thermodynamics. The PPI were estimated using the interaction potentials in the frame of DLVO theory and idealized molecular geometry. We have shown that they are in good agreement with the experimental data, thereby indicating the adequacy of this approach for modeling protein interactions in dilute and semi-dilute solutions [74,84,87].

**Figure 3:**
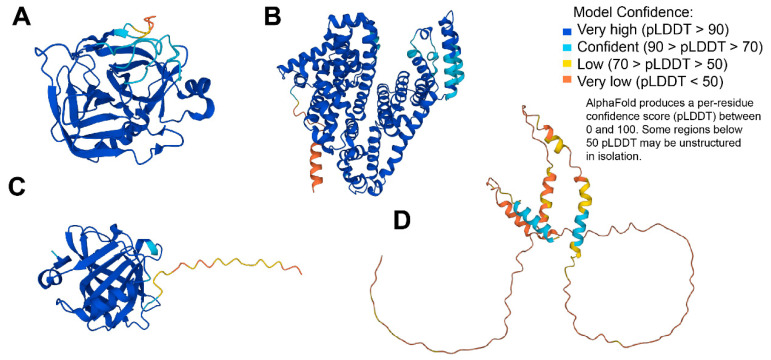
3D structures modeled for the discussed proteins by AlphaFold [88]: (**A**) α–chymotrypsin (UniProt ID: P00766); (**B**) human serum albumin (UniProt ID: P02768); (**C**) β-lactoglobulin (UniProt ID: P02754); (**D**) α-casein (UniProt ID: P02662).

In the present article, PPI were estimated using the examples of spheroidal proteins with various structural organization. They were two rigid globular proteins, α-chymotrypsin (ChTr) and human serum albumin (HSA), with spherical and ellipsoidal form, respectively; another two were spheroidal proteins α-casein (α-CN) and β-lactoglobulin (β-Lg) containing disordered fragments in their structure (see Figure 3). At low solution salinity, the dominance of electrostatic repulsion was shown for all studied proteins. However, α-CN and β-Lg exhibited the significant impact of van der Waals attraction in the total PPI potential, which was related to the tendency of these proteins to form associates. The increase of solution ionic strength resulted in the strong screening of the protein charges leading to decrease of electrostatic inter-protein repulsion for all proteins. The increase in the van der Waals attraction observed for the α-CN and ChTr was responsible for the ability of these proteins to form the short-living protein oligomers. At the same time, the increase in the ionic strength in the β-Lg solution caused the formation of stable oligomers, leading to the decrease in non-specific interactions.

## 2. Existing Theoretical Descriptions of Protein Translational Diffusion

The study of protein translational diffusion provides the unique way to reveal the intricacies of their inter-molecular interactions. The theory used for interpretation of experimental data is a key step for extracting such information. In the theoretical descriptions of the diffusion process, one can distinguish four levels:

1. Purely phenomenological models with accent on (a) hydrodynamics; (b) free volume theory; (c) effect of steric hindrances, etc. [89]. Such theories are constructed in an ad hoc manner for description of particular experiments. They usually stress only one, supposedly dominant type of particle interaction and neglect the others. The motivation of such approaches is mainly in the agreement of the corresponding fits with the experimental data rather than in the logical self-consistency of physical principles, which lie in their foundations. This highly superficial level of description was totally exhausted in ideas by the 2000s [89].

2. Semi-phenomenological approach based on the standard Stokes–Einstein formalism. It links the particle self-diffusion coefficient *D_s_* with the solute-solvent friction coefficient *f*_12_ (Equation (2)). This friction coefficient, in turn, is the function of solution viscosity *η* as well as the particle size and shape [90]. In this approach, the random collisions of Brownian particle with solvent molecules define diffusive character of the motion (i.e., the surrounding liquid medium provokes random particle displacements). In this approach, the friction coefficient *f*_12_ is a semi-phenomenological function of solution viscosity *η* and protein size. The dependence of *η* on molar concentration of the solute *C* is usually taken as a relationship [91]:

(5) $$\eta =\eta_{s}(1+[\eta ]C+k_{H}{\left[\eta \right]}^{2}C^{2}),$$

where *η_s_* is viscosity of pure solvent, [*η*] is the so-called intrinsic protein viscosity, and *k_H_* is the phenomenological parameter known as the Huggins coefficient (named after Maurice L. Huggins (1897–1981)), which is an indicator of the strength of a solvent that typically ranges from about 0.3 (for strong solvents) to 0.5 (for poor solvents).

3. Purely hydrodynamic models based on the stringent solution of corresponding Navier–Stokes equations for hard spheres [92], rod-like particles [93,94], etc. The case of hard spheres seems to be pertinent for spheroidal globular proteins. In this case, the self-diffusion coefficient *D_s_* is obtained as [92]:

(6) $$\frac{D_{s}}{D_{s}^{0}}=\frac{1-9\varphi /32}{1+H\left(\varphi \right)+(\varphi /\varphi_{0})/{\left(1-\varphi /\varphi_{0}\right)}^{2}},$$

where *φ*_0_ ≈ 0.5718 is the critical concentration of dense packing for hard spheres,

$$H\left(\varphi \right)=\frac{2b^{2}}{1-b}-\frac{c}{1+2c}-\frac{bc(2+c)}{\left(1+c)(1-b+c\right)}$$

$$b={\left(\frac{9}{8}\right)}^{1/2},c=\frac{11\varphi }{16}$$

The hard-sphere model yields quite reliable results (see, e.g., [38]). However, its applicability is extremely limited by spheroidal shape of particles and does not take into account various intermolecular interaction. Additional approaches are necessary for taking into account the effects of solute-solute and solute-solvent interactions on protein translational diffusion [95].

4. Finally, there is an approach proposed by Hans Vink [65]. It is based on the frictional formalism of non-equilibrium thermodynamics and provides a fundamental level of description for molecular diffusion. By now, it has become a well-established formalism for both self- and collective (or otherwise mutual) diffusion of various particles. The profound physical principles of non-equilibrium thermodynamics go back to the famous reciprocal relations for kinetic coefficients discovered by Lars Onsager (1903–1976) (see, e.g., [96]). On the one hand, the Vink’s approach makes use of the phenomenological solute–solute and solvent–solute hydrodynamic friction coefficients, and, on the other hand, it relates collective diffusion coefficient *D_s_* and solute virial coefficients, which include interaction potentials. As a result, the frictional formalism deals with the phenomenological coefficients, and their origin can be justified and revealed in molecular theories of more profound character. A notable characteristic of Vink’s approach is a clear-cut distinction between the self- and collective diffusion. The self-diffusion coefficient *D_s_* refers to the motion of a single particle in solution and depends both on the particle-particle and particle-solvent friction coefficients. As a result, *D_s_* characterizes the movement of solute particles relative to each other. In contrast, collective diffusion coefficient *D_c_* characterizes the flow of solvent molecules relatively to solute particles and, as a result, depends only on the particle-solvent friction coefficient. Therefore, the collective diffusion describes the movement of solute molecules past the solvent ones (the molecules of another type) while the self-diffusion describes the movement of solute molecules past themselves (i.e., actually a displacement of single molecule).

The Vink theory was successfully applied to several systems, such as non-associative fluorinated amphiphile [97], water solutions of non-ionic surfactants [98], charged block copolymers [99], wormlike micelles of non-ionic surfactants [100], amylopectin (homopolymer of D-glucose) [101], polysterene [102], rod-like polymers [103] in different solvents, and some other systems [54,104,105]. The dependence of Ds on the solute concentration was measured and interpreted within the framework of Vink theory for a number of globular proteins, including β-lactoglobulin [106], hemoglobin [107], serum albumin [26], ovalbumin [27,108], and lysozyme [109]. Additionally, it was applied to monoclonal antibodies [67,110], α-chymotrypsinogen [49], and proteins of various shape and size, such as chymotrypsin, α-casein, and fibrinogen [51,111,112].

For the self-diffusion coefficient of particle *D_s_*, the Vink theory yields:

(7) $$D_{s}=\frac{RT}{f_{12}c_{1}+f_{22}c},$$

where *f*_22_ and *f*_12_ are referred to the solute–solute and solvent–solute molar hydrodynamic friction coefficients, respectively, and *c* and *c*_1_ are the solute and solvent molar concentrations, respectively. For the normalized *D_s_*, one has

(8) $$\frac{D_{s}}{D_{0}}=\frac{f_{12}c_{1}}{f_{12}c_{1}+f_{22}c},$$

where *D*_0_ is the protein diffusion coefficient at infinite dilution.

Partial volume of the solvent molecule is denoted as *υ*_1_, and that of the solute as *υ*_2_. Then, the volume fraction of solute is *φ* = *cυ*_2_ and the analogous expression can be written for the solvent. The sum of solvent and solute volume fractions equals to 1:

(9) $$c_{1}\upsilon_{1}+c\upsilon_{2}=1,$$

If we denote:

(10) $$\rho =\frac{f_{22}\upsilon_{1}}{f_{12}\upsilon_{2}}\;\mathrm{and}\;\varphi =c\upsilon_{2},$$

Equation (10) can be rewritten as:

(11) $$\frac{D_{s}}{D_{0}^{}}=\frac{1}{1+\rho \frac{\varphi }{1-\varphi }}$$

On the other hand, for the collective diffusion coefficient *D_c_*, Vink’s formalism yields:

(12) $$\frac{D_{c}\left(\varphi \right)}{D_{0}}={\left(1-\varphi \right)}^{2}\left(1+\nu \varphi +\mu \varphi^{2}+\eta \varphi^{3}+\omega \varphi^{4}+\ldots \right)$$

where

(13) $$\nu =\frac{2\left|A_{2}\right|}{\upsilon_{2}};\mu =\frac{3A_{3}}{\upsilon_{2}^{2}};\eta =\frac{4A_{4}}{\upsilon_{2}^{3}};\omega =\frac{5A_{5}}{\upsilon_{2}^{4}}.$$

Here *A*_2_, *A*_3_, … are the second, third, etc., solute virial coefficients, respectively, in molar concentration units. They characterize the solute-solute (protein–protein, in our case) interactions. The second virial coefficient *A*_2_ is a valuation of pairwise interactions. However, if the solute concentration increases, there is inevitable need to introduce the multi-particle interactions, which are characterized by the higher-order virial coefficients.

In the course of our investigation attempts, we tried the hydrodynamic model of Michio Tokuyama and Irwin Oppenheim (type 3) in [68], the semi-phenomenological approach (type2) [113], and the Vink theory (type 4) [51,52]. In our opinion, the Vink’s approach gives the most profound and fundamental microscopic level of the description of diffusion coefficients for proteins with both regular (globular and cylindrical) or partially disordered structure.

## 3. Paired PPI Potential

The Vink’s formalism relates *D_c_* to the second virial coefficient *A*_2_, which is one of the most important PPI characteristics. Its value is determined by the combined action of various inter-molecule interactions, manifesting themselves in the potential of mean force *W*. The William G. McMillan–Joseph E. Mayer solution theory provides the relationship between *A*_2_ and *W* [114]:

(14) $$A_{2}(a_{1}^{0},T)=A_{2}^{hs}-\frac{N_{A}}{2}\int_{d+3\overset{\circ }{A}}^{\infty }\{\exp\left[-W(r,a_{1}^{0},T)-k_{B}T\right]-1\}4\pi r^{2}dr,$$

where $a_{1}^{0}$ is the activity of pure solvent, $A_{2}^{hs}=\left(2\pi d^{3}\right)/3$ is the hard-sphere contribution to *A*_2_, *d* is the diameter of protein molecule, and *r* is the radial coordinate. The lower limit of integration in (12) is chosen as *d* + 3*Å* to take into account a layer of water bound to the protein [81].

For protein molecules in solution, the pair interaction potential, *W*(*r*), is usually modeled within the framework of classical DLVO theory of the colloid suspension stability [115,116]. According to the DLVO theory, the total interaction potential is mainly determined by the sum of a long-ranged Coulomb potential and the van der Waals interactions [108,109]:

(15) $$W(r)=W_{el}(r)+W_{vdW}(r),$$

where *W_el_*(*r*)—electrostatic interaction potential, *W_vdW_*(*r*)—van der Waals interaction potential.

In our previous studies, several models of colloidal particles have been successfully applied for estimation of the interaction potential of proteins with different mass, shape, and structural rigidity [51,52]. We distinguished the model of porous colloid particle [117,118] as the most suitable in all cases, where protein molecules do not form associates in a rather wide concentration range [52]. The “porous” model represents the most complete description of protein charge shell including the surface charge distributions and the counter-ion layer Figure S1 (for calculation details see supplementary material). However, when we deal with the probable protein association, the “porous” model fails in description of experimental data [51]. For these cases, it is better to use the Yukawa electrostatic potential [119], which considers the effective ζ-potential as a charge characteristic of a protein molecule and may be used to obtain the satisfactory interpretation of the experimental results [51].

## 4. Self-and Collective Diffusion of Spheroidal Proteins

**Figure 4:**
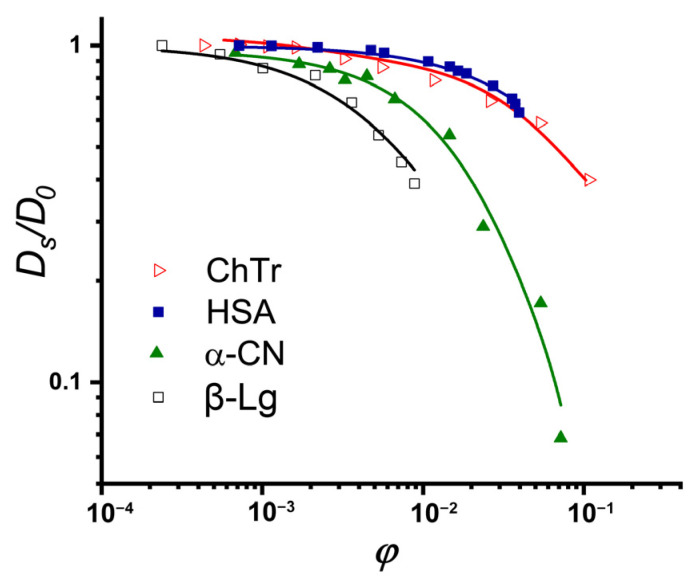
Normalized concentration dependencies of self- diffusion coefficients of ChTr (pH = 3.5, *I* = 0.01 M), HSA (pH = 7.2, *I* = 0.01 M), α-CN (pH = 7.0, *I* = 0.01 M), and β-Lg (pH = 7.0, *I* = 0.003 M) The original data were obtained from [51,73,120].

The concentration dependencies of the self-diffusion coefficients obtained with PFG NMR for ChTr, HSA, β-Lg, and α-CN are presented in Figure 4 [51,68,73,120]. The initial near-horizontal parts of the curves (Figure 4) characterize the region of the dilute solutions with the diffusion coefficients *D*_0_ of 15.2 · 10^−10^ m^2^/s, 9.63 · 10^−11^ m^2^/s, 8.65 · 10^−11^ m^2^/s, and 7.82 · 10^−11^ m^2^/s for ChTr, β-Lg, α-CN and has, respectively. We have estimated the protein hydrodynamic radii using the Stokes–Einstein equation (Equation (1)). These evaluations revealed that the *R_h_* values of the diffusing particles are 1.8 nm, 3.5 nm, 2.9 nm, and 3.2 nm for ChThasHSA, β-Lg, and α-CN respectively.

The preliminary analysis of the obtained concentration dependencies for protein self–diffusion coefficient shows that the diffusive mobilities of β-Lg and α_S_-CN are lower than those of the HSA and ChTr. The sharper, in comparison with the globular HSA and ChTr, concentration-dependent decrease in the α-CN self-diffusion is probably caused by the mostly disordered structure of α-casein molecule [68] (see Figure 3C). It is striking that β-Lg has the most precocious decrease of the diffusive mobility against its smallest molecular weight (18 kDa), whereas the presence of protein associates has not been proven. The reason for such early decrease of β-Lg self-diffusion is probably related with the partly disordered structure of β-Lg (see Figure 3B) and the presence of significant attractive PPI of β-Lg at low protein concentration [121,122,123].

**Figure 5:**
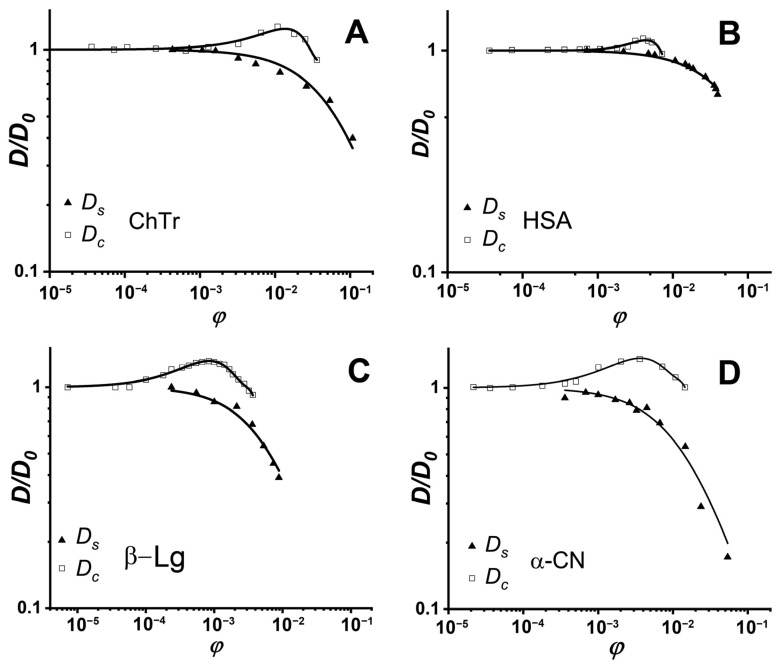
Normalized concentration dependencies of protein self- (triangles) and collective (squares) diffusion coefficients: (**A**) ChTr (pH = 3.5, *I* = 0.01 M); (**B**) HSA (pH = 7.2, *I* = 0.01 M); (**C**) β-Lg (pH = 7.0, *I* = 0.003 M); (**D**) α-CN (pH = 7.0, *I* = 0.01 M). Solid lines denote fits of experimental data by the Vink’s algorithm. The original data were obtained from [51,68,73,120].

We compared the translational diffusion coefficients obtained using the DLS and PFG NMR methods to get the information about weak intermolecular interactions. Figure 5 shows the concentration dependencies of ChTr, HSA, β-Lg, and α_S_-CN obtained by these two independent experimental methods, which observe different diffusion effects characterized by the self-diffusion coefficient *D_s_* for NMR and the collective diffusion coefficient *D_c_* for DLS. The estimation of the protein intermolecular interactions involves analysis of the translational diffusion using the methods that are sensitive to various molecular effects [51].

**Figure 6:**
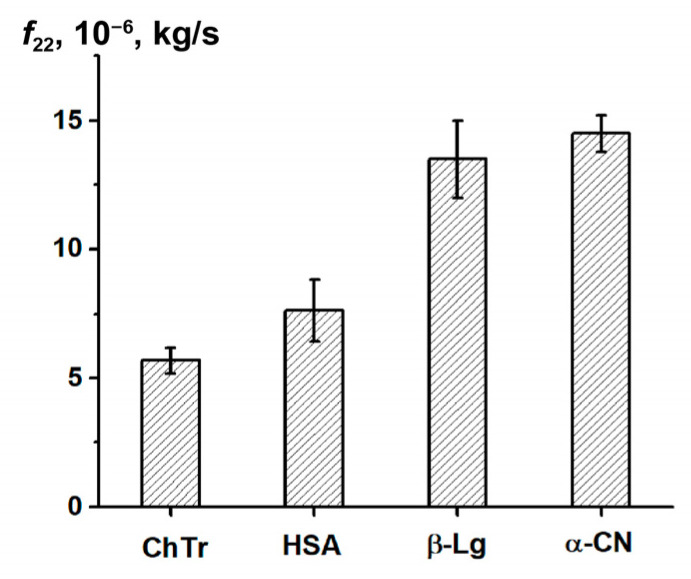
Protein–protein friction coefficient *f*_22_ for diluted protein solutions.

Earlier, Vink theory was successfully applied to the approximation of the experimental data on the self- and collective diffusion of proteins. It was shown that Vink theory well-described the experimental data obtained in the studied concentration range for spheroidal ChTr, HSA, β-Lg, and α-CN [51,68,73,120]. The numerical fitting of the experimental self- and collective diffusion data gives the friction and virial coefficients, respectively. According to the Vink’s approach, the hydrodynamic interactions are taken into account by introduction of the solvent–solute (*f*_12_) and the solute–solute (*f*_22_) friction coefficients. For dilute protein solutions, solvent–solute friction coefficient *f*_12_ can be determined by the Stokes–Einstein relation (Equation (2)). Using the thus retrieved *f*_12_ values and the fitting parameter *ρ*, the *f*_22_ values were calculated. The *f*_22_ value characterizes the influence of the direct and hydrodynamic interactions between the protein molecules on the protein self-diffusion [67,124]. Figure 6 shows *f*_22_ for ChTr, HSA, β-Lg, and α-CN in dilute solution (*φ* = 0.003). The *f*_22_ values for β-Lg and α-CN were found to be higher than those for ChTr and HSA. It can be associated with the influence of disordered fragments in β-Lg and α-CN structure, which can provide the steric PPI prior to the associates formation [125,126].

The collective diffusion coefficient and its approximation by the Vink’s algorithm (squares on Figure 5) made it possible to obtain the sets of virial coefficients (Table S1) [51,120]. Intermolecular interactions of participating partners manifest themselves in the number and values of virial coefficients. An analytical relationship between the experimental and theoretical values exists only for the second virial coefficient *A*_2_. This value contains information about pairwise intermolecular interactions, which are possible in dilute solutions. In the case of the semi-diluted and concentrated solutions, *A*_2_ cannot provide all information about protein interactions, since it is necessary to take into account the influence of the many-body interactions via the higher order virial coefficients.

The second virial coefficient *A*_2_ can be obtained using different experimental methods, such as dynamic light scattering (DLS) [65], static light scattering (SLS) [79], gas-chromatographic elution [127], and membrane osmometry [80] measurements. In previous works, the *A*_2_ values for ChTr, HSA, β-Lg, and α-CN were determined independently with DLS and SLS techniques [51,73,79,120,128]. Table 1 shows that the difference in *A*_2_ values of ChTr, has, and β-Lg obtained by two light-scattering methods is rather significant, which can be explained by the different protein environment in corresponding experiments. Furthermore, the difference of *A*_2_ values for α-CN by a factor of approximately 40 may be a result of the α-CN associate formation detected in the DLS experiment [128,129]. Therefore, the subsequent analysis of PPI for α-CN was based on the SLS data for α-CN monomers, and corresponding *A*_2_ values were obtained by Dickinson et al. [128]. For other proteins, the PPI estimations were based on the diffusion data from the DLS data combined with the Vink’s algorithm.

## 5. Paired PPI Potential of Spheroidal Proteins

**Figure 7:**
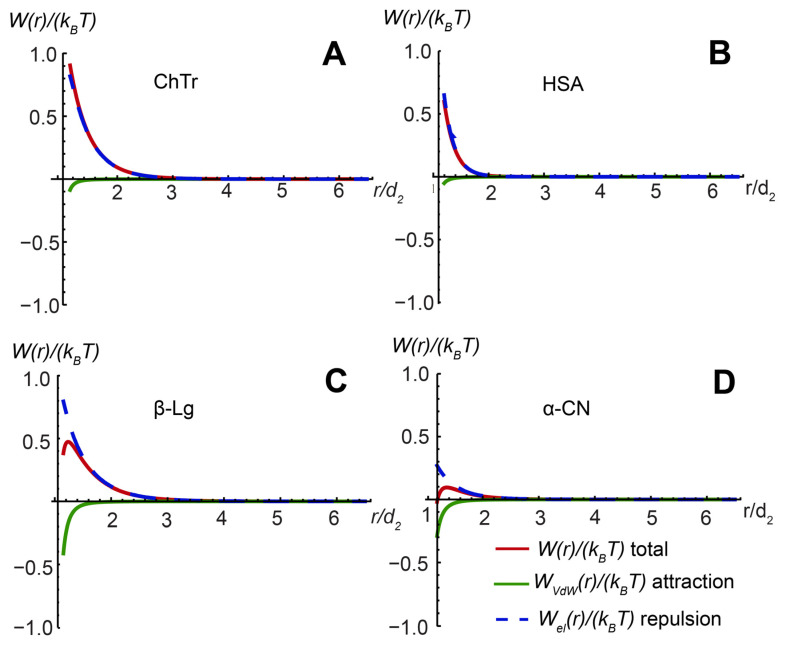
Total paired PPI potential and its constituent elements for (**A**) ChTr (pH = 3.5, *I* = 0.01 M); (**B**) has (pH = 7.2, *I* = 0.01 M); (**C**) β-Lg (pH = 7.0, *I* = 0.003 M); (**D**) α-CN (pH = 7.0, *I* = 0.01 M). The original data for ChTr ahasHSA were obtained from [51,120].

The second virial coefficient *A*_2_ is sensitive to the nature of “soft” PPI. The McMillan–Mayer theory (Equation (14)) is usually used for the quantifying of PPI, providing the relationship between the *A*_2_ and the total paired interaction potential *W* [131]. The effective interaction potential in the framework of the DLVO theory is represented by the attraction–repulsion balance between two molecules in solution and is determined by the contributions of electrostatic and van der Waals interactions (Equation (15)). The calculation of PPI potentials of spheroidal proteins ChTr, HSA, β-Lg, and α-CN was based on the model of spherical porous colloidal particle [117,132] (for calculation details, see Supporting Information). The corresponding data on the PPI potentials are presented in Figure 7. It was found that the main contribution to intermolecular interactions of all studied spheroidal proteins is made by electrostatic repulsion potential *W_el_*(*r*). However, in the cases of β-Lg and α-CN, the contribution of the van der Waals interaction was more noticeable. The stronger van der Waals potentials of β-Lg and α-CN are probably associated with the propensity of these proteins to self-associate [37,68,106,126,128]. The flexible disordered domains of β-Lg and α-CN can provide the attractive PPI potential during the associate formation. To create the favorable conditions for attractive interactions of proteins resulting in their association, it is necessary to reduce electrostatic repulsion. As a rule, for alteration of the electrostatic interactions, one can use the change in the ionic strength (i.e., the changes in the concentration of the free ions in solution).

## 6. Ionic Strength Influence on Repulsion–Attraction Balance in PPI

At low ionic strength (0.003 M–0.01 M), all spheroidal proteins have positive *A*_2_ values indicating the prevalence of the paired repulsive potential. The increase in the ionic strength (0.01 M–1.0 M) shows the strong charge screening reflected in the decrease in the Debye (screening) length *κ*^−1^ and negative value of *A*_2_ for ChTr, β-Lg, and α-CN (Table 2) [73,79,106,128]. It should be noted that for the rigid ChTr, a negative *A*_2_ is observed at a sufficiently high ionic strength (1.0 M). A negative value of *A*_2_ in the framework of DLVO theory characterizes the dominance of the van der Waals attractions [52,128,133,134]. Other factors affecting PPI, such as the steric ones, hydrogen bonding, and short-range hydration forces, are not included in the DLVO representation. These attractive effects can be considered as a correction to the van der Waals term by adjusting the Hamaker coefficient *H* [135]. A strong screening of protein charges leads to a significant probability of the neighboring protein molecules to stick and self-assemble, which is expressed in the increasing values of Hamaker constant (Table 2).

**Figure 8:**
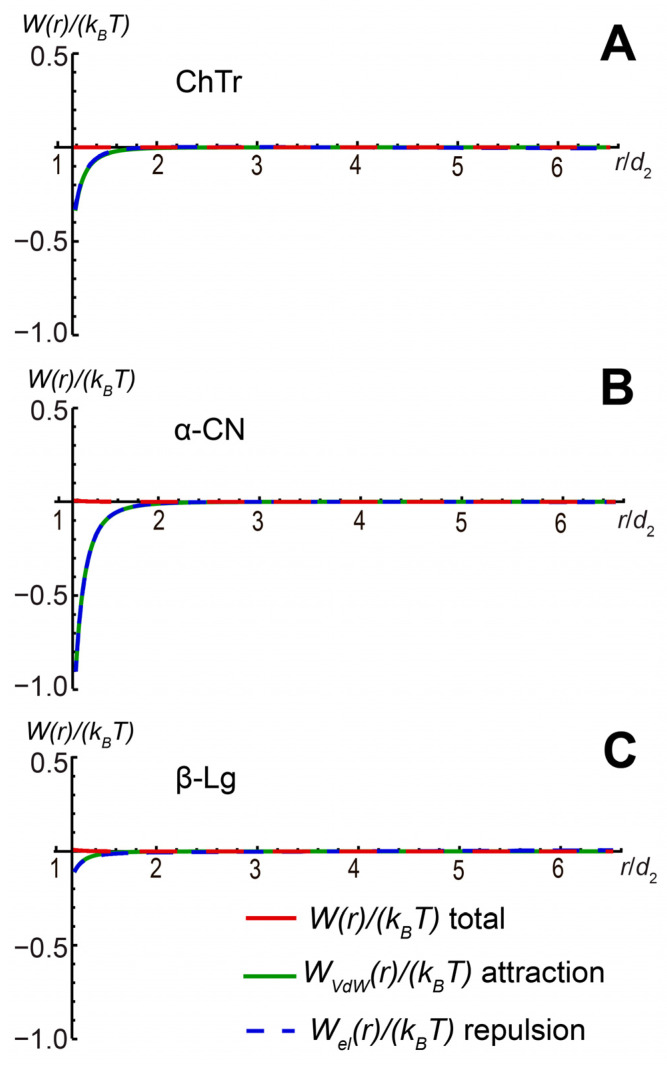
Total paired PPI potential and its contributing parts for (**A**) ChTr (pH = 3.5, *I* = 1.0 M); (**B**) α-CN (pH = 7.0, *I* = 0.1 M); (**C**) β-Lg (pH = 7.0, *I* = 0.1 M).

Finally, using the protein–protein second virial coefficient *A*_2_ at increasing ionic strength, we estimated the contributions of the electrostatic and van der Waals interactions to the total paired PPI potential *WI* (for calculation details, see Supplementary Information). Our results show that the increase in the salinity of the protein solutions associated with a strong screening of protein charges results in the significant decrease of the electrostatic repulsion and the dominance of the protein–protein attraction (Figure 8).

The β-Lg and α-CN self-association is highly dependent on the ionic strength (*I*) of the solution [68,73,125,126,129]. Furthermore, β-Lg at *I* = 0.1 M could form stable oligomers, leading to a decrease in the resulting weak non-specific PPI. In our opinion, the van der Waals attraction of protein molecules contributes to the further self-association of proteins. This effect is especially pronounced for α-CN. The main reason for this behavior is likely the non-electrostatic interactions between disordered fragments of its molecules. However, these intra-molecular interactions are relatively weak and unstable in solution. With a further increase in the salinity or due to other favorable factors, these attractive interactions lead to the formation of stable protein self-associates, as was observed for β-Lg [136,137].

## 7. Conclusions

PPIs have a pivotal role in biological processes in living systems, controlling and modulating the direction protein functioning, such as, for example, signal transduction, associated with various diseases, including cancer, infections, and neurodegenerative diseases [138].

In the present article, we analyzed the uniform approach to study intermolecular interactions of proteins in solutions. This approach is based on the analysis of the translational diffusion data. It was applied to a set of the spheroidal proteins differing in degree of structural (dis)order. The reviewed approach carries out the inter-complementary analysis of the protein self- and collective diffusion coefficients obtained by the experimental methods of nuclear magnetic resonance with pulsed gradient of magnetic field (PFG NMR) and spectroscopy of dynamic light scattering (DLS). The combination of concentration dependencies for coefficients of self- and collective diffusion with the Vink theory (phenomenological approach based on the formalism of non-equilibrium thermodynamics) enables one to obtain the sets of friction and virial coefficients for proteins studied. The second and higher virial coefficients were obtained for estimation of pair and multi-particle intermolecular interactions in solutions with low values of the ionic strength (0.003–0.01 M) for ChTr, HSA, α-CN, and β-Lg. The McMillan–Mayer theory can be used for quantitative estimation of the non-specific PPI. This theory provides the relationship between the second virial coefficient *A*_2_ and the effective potential of paired interactions *W*(*r*) within the framework of DLVO theory. In this theory, the balance of attraction-repulsion interactions between the two protein molecules in solution depends on the electrostatic and van der Waals potentials. The positive value of the second virial coefficient *A*_2_ for spheroidal ChTr, HSA, α-CN, and β-Lg at low ionic strengths (0.003–0.01 M) means the dominance of the intermolecular repulsion. The increase in ionic strength (0.1–1.0 M) led to the screening of the protein charges and, as a result, to the decrease in the electrostatic potential. The increase in the van der Waals potential for ChTr and α-CN can explain the propensity of these proteins to weak unstable attractive interactions. The decrease in the strength of the van der Waals interaction for β-Lg is probably associated with oligomers formation.

## Figures and Tables

**Table 1:** Second virial coefficients of proteins obtained by the light scattering [51,73,79,120,128,130].

	*A*_2_,·10^−4^ m^3^ mol/kg^2^ (DLS)	*A*_2_,·10^−4^ m^3^ mol/kg^2^ (SLS)
ChTr	4.96 ± 0.08	3.8
HSA	0.46 ± 0.1	1.5
β-Lg	163 ± 6.5	42
α-CN	105 ± 7	2.7

**Table 2:** Second virial coefficient *A*_2_, Debye screening length *κ*^−1^, Hamaker constant *H* of ChTr, β-Lg, and α-CN at various ionic strength *I* values [73,79,106,128].

	*I* = 0.003–0.01 M				*I* = 0.1–1.0 M		
	*A*_2_,·10^−4^ m^3^ mol/kg^2^	*κ*^−1^, nm	*H*, *k_B_T*		*A*_2_,·10^−4^ m^3^ mol/kg^2^	*κ*^−1^, nm	*H*, *k_B_T*
ChTr (0.01 M)	4.96	3.04	1.1	ChTr (1.0 M)	−0.44	0.3	6
β-Lg (0.003 M)	16	6.52	1	β-Lg (0.1 M)	−1	0.98	5
α-CN (0.01 M)	2.7	3.04	5	α-CN (0.1 M)	−20.6	0.78	15

## Data Availability

The data in this study are available on reasonable request from the corresponding author.

## Supplementary Materials

The following supporting information can be downloaded at: https://www.mdpi.com/article/10.3390/ijms23169240/s1.
