# Supplementary material for: Effect of Protein–Protein Interactions on Translational Diffusion of Spheroidal Proteins
Source: Int J Mol Sci. 2022 Aug 17;23(16):9240. doi: 10.3390/ijms23169240 (PMC9409276; doi:10.3390/ijms23169240)
Supplement: Supplementary file 1 [file ijms-23-09240-s001.zip › ijms-1766274-supplementary.pdf]

Supporting Information

Effect of protein-protein interactions on translational diffusion of spheroidal proteins

Aleksandra M. Kusova [1], Aleksandr E. Sitnitsky [1], Vladimir N. Uversky [2] and Yuriy F. Zuev [1*]

[1] Kazan Institute of Biochemistry and Biophysics, FRC Kazan Scientific Center, Russian Academy of Sciences, Lobachevsky Str., 2/31 Kazan, 420111, Russia

[2] Department of Molecular Medicine and Byrd Alzheimer's Research Institute, Morsani College of Medicine, University of South Florida, 12901 Bruce B. Downs Blvd., Tampa, FL 33612, USA

* Correspondence: yufzuev@mail.ru; Tel.: +7-(843)-2319036

1. Electrostatic and van der Walls potentials: computational details

It is common practice to use colloid theories to represent various physicochemical properties of proteins, and so we applied the well-developed theory of "porous" colloid particles of different shapes (Ref. 117). In an electrolyte solution, a charged protein molecule interacts with the mobile counter-ions. The tendency to neutralize the surface charges of particles is counteracted by the thermal motion of these ions. As a result, an ionic cloud of mobile counter-ions is formed around the charged particle.

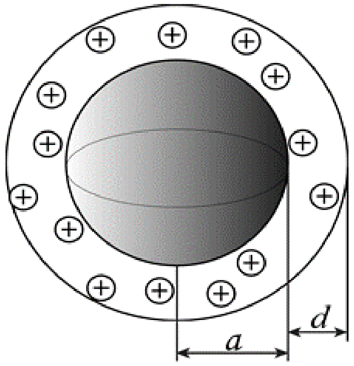

Figure S1. Model representation of spherical particle with core (dark) and ion-penetrable surface layer.

For porous particles, the distance between geometrical (mass) centers of particles, r, is related to distance between the particle surfaces h as

$$h = r - 2d - 2a, \quad (1)$$

where d is the thickness of ion-penetrable surface layer and a is the radius of particle core.

Taking into account the effect of electric charge and ionic environment of spheroidal ChTr the electrostatic interaction potential of two "porous" spherical particles can be written as (Ref. 117):

$$W_{el}(r) = 4\pi\varepsilon_r\varepsilon_0 a^2\psi_0^2 \frac{e^{-\kappa(r-2d-2a)}}{r}, \quad (2)$$

where a is the sphere radius, r is the radial distance, ε_0 is the permittivity of vacuum, ε_r is the relative permittivity;

κ is the inverse Debye screening length:

$$\kappa^{-1} = \sqrt{\frac{\varepsilon_r\varepsilon_0 k_B T}{q_{ion}^2 e^2 \rho_\pm}}, \quad (3)$$

here k_B is the Boltzmann constant, T is the temperature, e is the electron charge, q_{ion} is the ion valence, $\rho_\pm$ is the bulk concentration of ions in suspension;

ψ_0 is the unperturbed surface potential (i.e., the potential of an isolated molecule) of a "porous" sphere:

$$\psi_0 = \frac{\rho_{fix}}{2\varepsilon_r\varepsilon_0\kappa^2}\left(1+e^{-2\kappa a}-\frac{1-e^{-2\kappa a}}{\kappa a}\right), \quad (4)$$

where ρ_{fix} is the charge density of the surface charged layer, which is given by:

$$\rho_{fix} = \rho_V + \sum_i z_i e \rho_{0i}. \quad (5)$$

In Eq.(5) ρ_v is the protein volume charge density, e is the electron charge, z_i is the valence and $\sum_i \rho_{0i}$ is the concentration of ions at the surface, which is given by the Grahame equation (Ref. 116):

$$\sum_i \rho_{0i} = \sum_i \rho_{\infty i} + \frac{\sigma^2}{2\varepsilon_0\varepsilon_r k_B T}, \quad (6)$$

where σ is the surface charge density, $\sum_i \rho_{\infty i}$ being the ionic concentration in the bulk. The potential of the van der Waals interactions of spherical particles is given as:

$$W_{vdw}(r) = -\frac{H}{6}\left\{\frac{2a^2}{r^2-4a^2}+\frac{2a^2}{r^2}+\ln\left(1-\frac{4a^2}{r^2}\right)\right\}, \quad (7)$$

where H is the solute Hamaker constant.

2. Characteristics of the studied protein systems

For PPI potentials estimation the following protein characteristics were used: diameter of molecule is 3.32 nm, 5.7 nm, 5.63 nm and 6.42 nm for ChTr, HSA, β-lg and α-CN (determined on the diffusion coefficient in dilute solution); protein permittivity is εs = 5; net protein charge is + 4.5e for ChTr, -23e for HSA, -9 for β-lg, -21.3e for α-CN, protein charge was determined on the basis of the zeta potential. The Debye length κ^{-1} and ρ_{fix} were determined depending on the parameters of the buffer solution according to (Ref. 116).

Table S1. Virial coefficients obtained from DLS experiment for ChTr (pH =3.5, I =0.01M), HSA (pH =7.2, I =0.01M), β-lg (pH =7.0, I =0.003M) and α-CN (pH =7.0, I =0.01M).

	$A_2 \cdot 10^{-4}$, m^3mol/kg^2	$A_3 \cdot 10^{-5}$, m^6mol/kg^3	$A_4 \cdot 10^{-6}$, m^9mol/kg^4	$A_5 \cdot 10^{-7}$, $m^{12}mol/kg^5$
ChTr	4.96±0.08	–0.8±0.017	0.035±0.00015	-
HSA	0.46±0.1	-0.54±0.1	8.5±0.71	-8.2±0.5
β-lg	163±17	-640±80	982±123	1490±345
α-CN	105±7	–280±50	300±80	–120±5
